# Interaction of Polymer of Intrinsic Microporosity PIM‑1 with Explosive Analytes at the Molecular Level: Combined Experiment and Computational Modeling

**DOI:** 10.1021/acs.jpcc.5c08293

**Published:** 2026-04-18

**Authors:** Salam Mohammed, Edward B. Ogugu, Ramakant Sharma, Dominic Taylor, Graeme Cooke, Neil B. McKeown, Glib Baryshnikov, Hans Ågren, Ifor D. W. Samuel, Graham A. Turnbull

**Affiliations:** † Swedish EOD and Demining Centre—SWEDEC, Swedish Armed Forces, SE-575 28 Eksjö, Sweden; ‡ Organic Semiconductor Centre, School of Physics and Astronomy, 7486 University of St Andrews, St Andrews KY16 9SS, U.K.; § School of Chemistry, 3124 University of Edinburgh, Edinburgh EH9 3FJ, U.K.; ∥ School of Chemistry, University of Glasgow, Glasgow G12 8QQ, U.K.; ⊥ Department of Science and Technology (ITN), 4566 Linköping University, SE-581 8 Linköping, Sweden; # X-ray Photon Science, Department of Physics and Astronomy, Uppsala University, Box 516, 751 20 Uppsala, Sweden; ∇ Faculty of Chemistry, Wroclaw University of Science and Technology, Wyspiańskiego 27, PL-50370 Wroclaw, Poland

## Abstract

This work investigates the molecular-level interactions between fluorescent microporous polymer PIM-1 and nitroaromatic explosives relevant to thin-film sensing. Thin films of PIM-1 were exposed to 2,4-dinitrotoluene (DNT) and 2,4,6-trinitrotoluene (TNT), and changes in the steady-state absorption and emission spectra were measured. Responses to nonexplosive aromatics such as benzene were also evaluated for comparison. Complementary electronic-structure calculations predicted optical spectra and determined binding energies of the PIM-1–analyte complexes. The results agree with the experiment and show that the association of nitroaromatic molecules alters PIM-1 energy levels and frontier orbital arrangements, indicating significant electronic interactions that enable photoexcited electron transfer. While the excited-state properties can be modeled using a single polymer repeat unit, binding requires at least three units, with DNT/TNT accommodated into a pocket in the contorted polymer backbone. These combined insights help us understand the molecular sensing interaction in PIM-1 for selective nitroaromatic detection and may help guide molecular design for binding interactions that enhance future sensor development.

## Graphical abstract

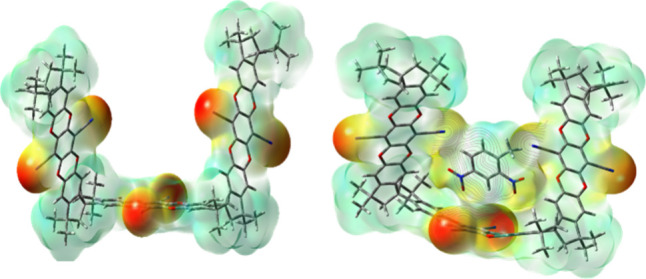

## Introduction

The detection of trace amounts of hazardous chemicals presents a significant and critical challenge across a variety of fields, including homeland security, environmental monitoring, and humanitarian landmine clearance efforts.^1^ These applications require highly sensitive and reliable methods for identifying harmful substances at very low concentrations, particularly in complex environments where conventional detection techniques may be less effective.^2,3^ Among the most promising approaches for achieving this level of sensitivity are fluorescent polymer sensors, which capitalize on an optical quenching mechanism activated in the presence of vaporized explosive molecules such as TNT.^4–6^ These sensors provide an attractive solution for detecting concealed explosives, chemical residues, or forensic traces, offering a remarkable level of sensitivity and specificity that is essential in applications where every detection is vital to safety and security.^7,8^

The fundamental principle behind the operation of these fluorescent polymer sensors is the fluorescence quenching process.^9^ This process occurs when a photoexcited polymer chain transfers an electron to an adjacent nitroaromatic molecule, such as those present in explosive vapors.^10^ This electron transfer leads to a reduction in light emission from the sensor film, which is the basis for detection. As a result, the system operates on a molecular scale, enabling the detection of even extremely low concentrations of hazardous chemicals.^11^ When explosive molecules are present in the vapor surrounding the fluorescent polymer film, they are first absorbed into the polymer matrix. Once absorbed, these molecules can influence the sensor’s optical properties in several ways.^12^ For example, the aforementioned change in the fluorescence intensity, and also a change in fluorescence wavelength, or they may alter the optical absorption spectrum, which can be measured using colorimetric sensing techniques.^13^

One of the key features that enhances the sensitivity and speed of detection in these systems is the strong noncovalent interactions between the fluorophore film and the absorbed analyte molecules.^11^ These interactions can lead to the accumulation of even weak vapor concentrations from the headspace above the sensor, which effectively increases the sensor’s response.^14^ As a result, the detection of hazardous chemicals becomes faster and more sensitive, even in environments with low vapor concentrations. Additionally, these interactions can help provide a distinctive signature for specific analytes, enabling selective detection of particular explosives or chemicals when the explosive molecule is desorbed from the fluorescent polymer film.^15^ This ability to selectively identify specific analytes further enhances the value of fluorescent polymer sensors in sensitive and high-stakes applications, such as bomb detection or environmental monitoring.

Polymers of intrinsic microporosity (PIMs) were originally coined and developed by one of the present authors.^16^ They represent a promising class of materials that have garnered significant attention due to their unique structural properties and potential applications in sensing technology.^17,18^ PIMs are macromolecules with highly contorted, rigid molecular structures, which create an amorphous, porous network on the nanometer scale.^19^ This structure allows them to possess a high surface area and high gas permeability, making them ideal candidates for use in gas separation and filtration systems.^20,21^ More importantly, the ability to control molecular interactions through their molecular design is a key feature that makes PIMs particularly attractive for advanced sensing applications.^6^

Certain PIMs are fluorescent, which provides an opportunity to significantly enhance fluorescence-based sensing techniques. By carefully tailoring the design of these materials, it may be possible to create sensors with optimized sensitivity, speed, and selectivity for a range of chemical threats. For example, their high surface area and tunable porous structure allow for rapid analyte absorption, which accelerates the overall detection process. Furthermore, by adjusting the molecular design, it is possible that PIMs may offer routes to enhance the polymer’s interaction with specific analytes, enabling the development of selective sensors that can differentiate between different chemical compounds.^22^ It is therefore very important to better understand the interaction between the PIM matrix and analyte molecules of interest in at the molecular scale. To this end, we combine spectroscopic measurements and ab initio calculations of the interaction of the prototypical polymer PIM-1 and common nitroaromatic molecules.

In the present work, we show that the interaction between PIM-1 and explosive analytes (DNT and TNT) causes a spectral shift in absorption and strong quenching of the fluorescence due to the photoexcited electron transfer in the bound donor–acceptor complex. We visualize this process within the density functional theory (DFT) simulations to confirm a photoexcited electron transfer to the analyte. Furthermore, we estimate the binding energy experimentally from the thermal release of bound analyte molecules at elevated temperature. We find that the noncovalent binding of DNT and TNT to PIM-1 can be predicted by DFT simulations and involves an electrostatic interaction with multiple repeat units of the polymer.

## Methods

PIM-1 was synthesized by the double nucleophilic aromatic substitution reaction between 5,5′,6,6′-tetrahydroxy-3,3,3′,3′-tetramethyl-1,1′-spirobisindane and tetrafluoroterephthalonitrile in the presence of potassium carbonate as a base.^23,24^ This yielded a bright yellow powder that was purified by reprecipitation from chloroform solution into methanol: the purity of the sample was confirmed by NMR spectroscopic characterization and gel permeation chromatography (see Section SI 1).

Microporous films of PIM-1 were fabricated by spin-coating a 20 mg/mL PIM-1 solution in chloroform at 2000 rpm for 60 s on 12 mm diameter fused silica substrates (UQG optics), resulting in a film thickness of approximately 180 nm, as measured by spectroscopic ellipsometry. Prior to spin-coating, the substrates were cleaned ultrasonically for 10 min in acetone, followed by isopropanol, then dried in a nitrogen stream, and plasma-ashed in 100% oxygen (Plasma Technology MiniFlecto) for 3 min. The PIM-1 films were subsequently doped with explosive analytes DNT and TNT by drop-casting 20 μL of 1 mM solutions in acetonitrile onto the polymer films, yielding masses of 3.64 and 4.45 μg for DNT and TNT, respectively. The solutions were allowed to evaporate, leaving behind molecules of analytes adsorbed in the films before steady-state measurements. The response of PIM-1 to nonexplosive molecules was also explored by drop-casting 20 μL of benzene (BN) and a control of 20 μL of only the acetonitrile solvent.

UV–vis absorption (using Jasco 770 UV–vis–NIR spectrophotometer) and fluorescence spectra (using Edinburgh Instruments FS5) of the films were measured before and after analyte exposure and also after thermal desorption of the sorbed analyte. The thermal desorption data were used to estimate the binding energy of DNT, as discussed in the Results and Discussion section. For this experiment, the PIM-1 film was loaded with 16.39 μg of DNT (giving an initial volume fraction of DNT of 14%) and heated using a hot plate for 3 min at each of a series of temperatures starting from 40 °C and increasing in steps of 10 °C. We believe that the loaded 16.39 μg of DNT is sorbed into the PIM-1 film, as polymeric materials are known to swell, allowing analyte molecules to be sorbed into the thin films.^25,26^ The thermal desorption is largely from the bulk of the PIM-1 film. The sample was heated in a fume hood to enable safe extraction of DNT vapors from the lab. After each heating step, an absorption measurement was made to determine the amount of the analyte lost. The film was then removed from the spectrophotometer and heated at a higher temperature before the next absorption measurement, and the process was repeated up to 200 °C. The characteristic optical responses arise from differences in polymer–analyte binding and photoinduced electron transfer quenching. To relate these processes to sensor response, we made a combined experimental and theoretical study of explosives sensing using the microporous polymer PIM-1.

A molecular model of PIM-1 (Figure 1a) was used for calculations of optical properties of absorption and emission, represented for electronic level calculations by a single repeat unit 1U-PIM-1 of the polymer (Figure 1b). Thus, the spiro-center and methyl groups of PIM-1 have been omitted and the terminal substituted by a hydrogen atom.^27^ However, to accurately calculate the binding energy (Δ*E*), we included the contorted PIM-1 structure with multiple repeat units of up to ten oligomer units (10U-PIM-1) of PIM-1. Full geometry optimization for both isolated 1U-PIM-1 and PIM-1 complexes (1U-PIM-1 + DNT, 1U-PIM-1 + TNT, and 1U-PIM-1 + BN) was then performed at the density functional theory (DFT) level, using the hybrid B3LYP exchange–correlation functional^28^ in conjunction with the 6-31G­(d) basis set for all cases (Figure 1a–e and Section SI 2). All calculations were performed using the Gaussian 16 program.^29^ The absorption spectra and fluorescence emission characteristics of 1U-PIM-1 and PIM-1 complexes with analyte molecules (DNT, TNT, and BN) were calculated by using time-dependent (TD) DFT^30^ at the B3LYP/6-31G­(d) level. However, for the calculations of Δ*E*, we applied B3LYP-gCP-D3/6-31G­(d), since the B3LYP functional has known limitations in accurately modeling noncovalent interactions like dispersion forces^31–35^ (see the comparison between the two approaches in Sections SI 10–SI 15).

**Figure 1:**
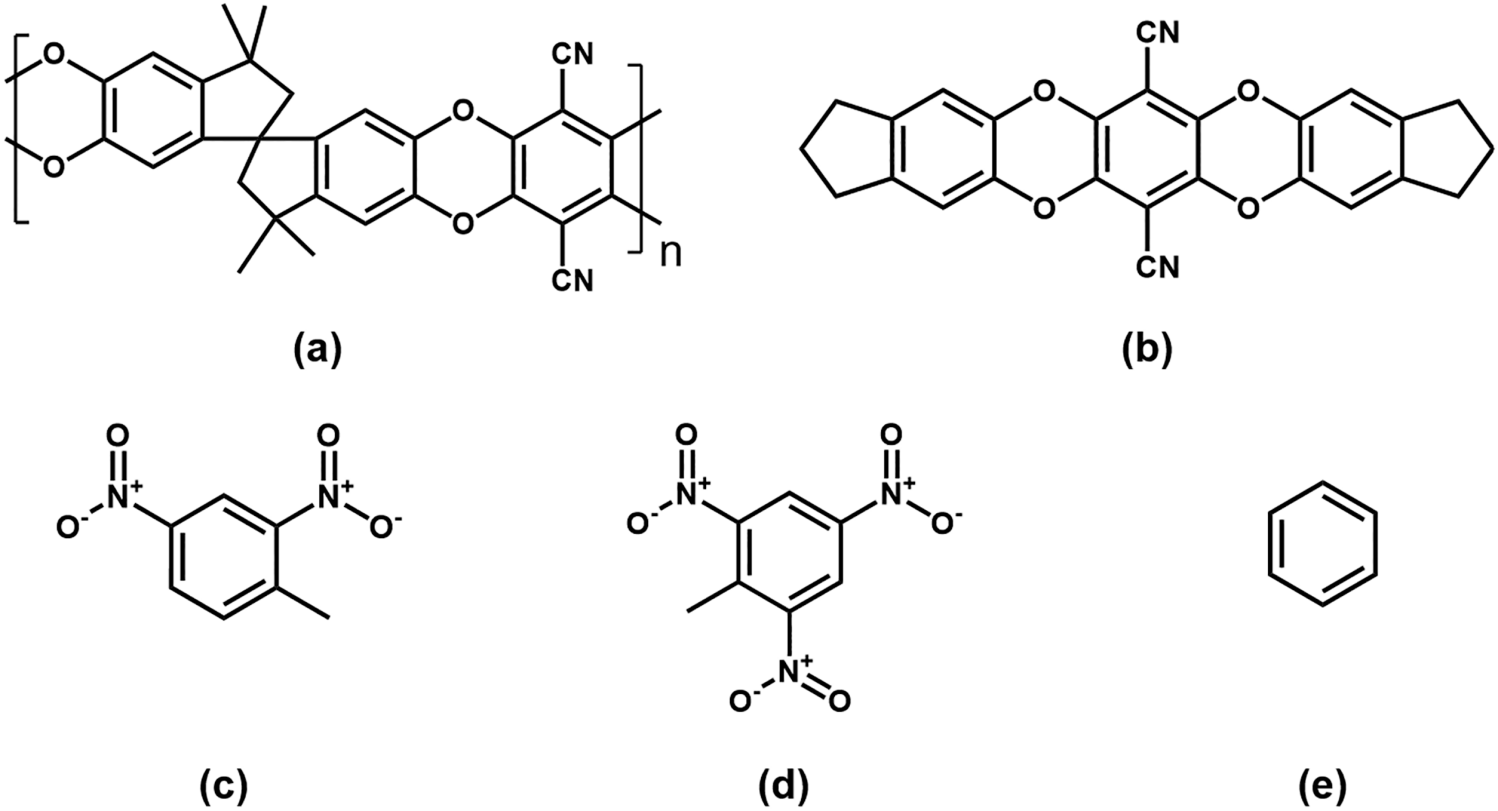
Molecular structure of (a) PIM-1. (b) Molecular model of the fragment of one unit of PIM-1 (1U-PIM-1), (c) DNT, (d) TNT, and (e) BN.

The optimized Δ*E* values between a DNT molecule and four different oligomeric configurations of PIM-1 (1U-PIM-1, 2U-PIM-1, 3U-PIM-1, and 10U-PIM-1) were also investigated using B3LYP-gCP-D3/6-31G­(d). The Δ*E* between XU-PIM-1 and DNT is defined as

ΔE=E(XU‐PIM‐1+DNT)−[E(XU‐PIM‐1)+E(DNT)] 1

where *E*(XU-PIM-1 + DNT), *E*(XU-PIM-1), and *E*(DNT) represent the energy of the XU-PIM-1 + DNT, XU-PIM-1, and the DNT molecule, respectively.

In order to estimate the corresponding experimental desorption energy, *E*_d_, during the stepwise thermal desorption of DNT from the PIM-1 film, we apply a modified Arrhenius equation to the experimental data. Herein, we derive the modification starting from the Arrhenius equation:^36^

$$k=k_{0}\;e^{-E_{a}/RT}$$ 2

where *k* is the specific reaction rate, *k*_0_ is the frequency or pre-exponential factor, *T* is the absolute temperature, *R* is the ideal gas, and *E*_a_ is the activation energy. The absorbance, *A*, is proportional to the concentration of quenchers [*Q*(*t*)], which follows the time dependence:

$$\frac{d[Q(t)]}{dt}=-k[Q(t)]$$ 3

The solution to eq 3 is

$$[Q(t)]=[Q(t=0)]\;e^{\left(-kt\right)}$$ 4

We assume that the film is heated at a specific temperature during time interval (*t*_0_ → *t*_0_ + Δ*t*). For each such heating step:

$$[Q(t_{0}+\Delta t)]=[Q(t_{0})]\;e^{\left(-k\Delta t\right)}$$ 5

$$\frac{\left[Q(t_{0})\right]}{\left[Q(t_{0}+\Delta t)\right]}=\frac{A(t_{0})}{A(t_{0}+\Delta t)}=e^{\left(k\Delta t\right)}$$ 6

Thus, the specific reaction rate is given by

$$\frac{1}{\Delta t}\ln\left(\frac{A(t_{0})}{A(t_{0}+\Delta t)}\right)=k$$ 7

Substituting *k* from eq 2 into eq 7 gives

$$\frac{1}{\Delta t}\ln\left(\frac{A(t_{0})}{A(t_{0}+\Delta t)}\right)=k_{0}\;e^{-E_{d}/RT}$$ 8

$$\ln\left\{\ln\left(\frac{A(t_{0})}{A(t_{0}+\Delta t)}\right)\right\}=-\frac{E_{d}}{RT}+\ln(\Delta tk_{0})$$ 9

For a series of heating steps of increasing temperature, each heating step of the series can be written as

$$\ln\left\{\ln\left(\frac{A(t_{i})}{A(t_{i}+\Delta t)}\right)\right\}=-\frac{E_{d}}{RT}+\ln(\Delta tk_{0})$$ 10

In the present work, we use eq 10 to estimate the desorption energy, *E*_d_.

## Results and Discussion

The experimental and theoretical absorption and emission spectra for PIM-1 and the PIM-1 complex (PIM-1 + DNT, PIM-1 + TNT, and PIM-1 + BN) are presented in the upper and lower panels of Figure 2, respectively, and solution absorption spectra of DNT and TNT are shown in Section SI 3. The experimental UV–vis spectra for these four cases (PIM-1, PIM-1 + DNT, PIM-1 + TNT, and PIM-1 + BN) are dominated by three broad structureless absorption bands with a maximum around 240 nm and one comparatively weak shoulder to the main peak around 300 nm and a slightly broader band around 430 nm, respectively. In the presentation of the theoretical spectra (of 1U-PIM-1, 1U-PIM-1 + DNT, 1U-PIM-1 + TNT, and 1U-PIM-1 + BN), we have included bars to represent the oscillator strengths as well as provided a line profile obtained by an application of a Gaussian line broadening. Both the experimental measurements and the theoretical calculations show that the bands around 240 and 300 nm for all cases overlap without a significant change in the spectra from addition of the analyte. We note, however, that the experimental spectra for the band around 430 nm is red-shifted by analyte addition of DNT (PIM-1 + DNT) and TNT (PIM-1 + TNT), causing a shift about 10 nm compared to the isolated PIM-1 and PIM-1 + BN (upper inset). There was no spectral change observed after drop-casting the acetonitrile solvent alone, indicating that the addition of TNT or DNT is responsible for the red shift. These spectral changes were also predicted by the DFT calculations (lower inset), which shows a compelling overall agreement between the theoretical and experimental spectra. Red-shifted absorption bands may arise due to Meisenheimer complex formation with nitroaromatic molecules, although we think this is unlikely in the current case without the presence of a strong nucleophile. The simulations show that the red shift can arise from a noncovalent donor–acceptor association of the polymer and analyte.

**Figure 2:**
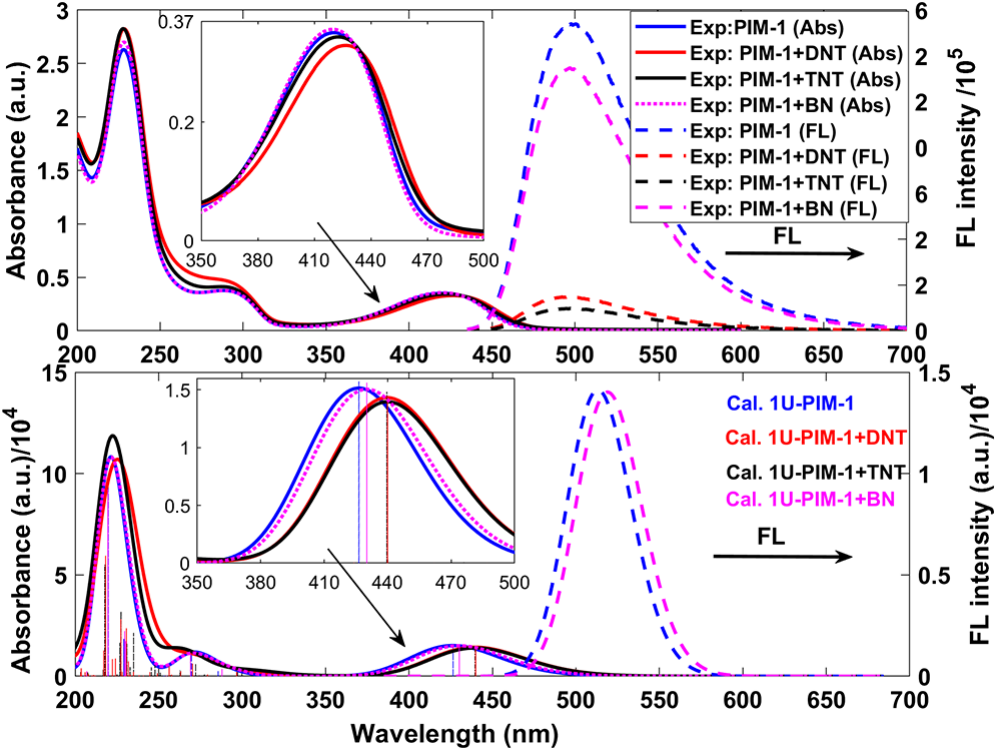
Experimental (upper panel) and calculated (lower panel) absorption spectra (solid/dotted lines) and emission spectra (dashed lines) of the investigated molecules. The calculated spectra of 1U-PIM-1, 1U-PIM + DNT, 1U-PIM-1 + TNT, and 1U-PIM-1 + BN (Section SI 2) are based on the electronic oscillator strength distribution, broadened by Gaussian line profiles. The insets show expanded measured and calculated spectra in the wavelength between 350 and 500 nm, respectively.

The experimental and theoretical calculations of the emission spectra for PIM-1 and PIM-1 complexes (PIM-1 + DNT, PIM-1 + TNT, and PIM-1 + BN) are also presented in Figure 2 in the wavelength region between 450 and 700 nm. Exposure to DNT or TNT leads to a strong quenching of the fluorescence that changes with the concentration of the nitroaromatic analyte sorbed in the film but without a significant change in the spectrum (Section SI 4). It is well-known that the observed luminescence is a result of recombination of the singlet exciton state. Specifically, excited, delocalized π electrons (π*) transition to the ground state (π), releasing energy as light. However, interactions between the CN group of PIM-1 and the nitroaromatic analyte (see Section SI 5) can give rise to photoinduced electron transfer to the nitroaromatic molecule, competing with the radiative transition and hence leading to a reduction in luminescence. The TD-DFT calculations provide additional visualization for this process in electronic spectra of the PIM-1 complex. The presence of the nitrate in, e.g., DNT makes it electron-deficient so that when the DNT is in contact with the electron-rich PIM-1, photoinduced electron transfer occurs between the lowest unoccupied molecular orbital (LUMO) of the polymer and the LUMO of the analyte molecule with a lower energy state. As shown in Figure 3a, the LUMO orbital level of 1U-PIM-1 is −2.20 eV, which is higher than the corresponding LUMO of the DNT (−3.00 eV) or TNT (−3.50 eV). This may suggest that the excited electrons on the LUMO orbital of 1U-PIM-1 could transfer to the LUMO of, e.g., DNT, and, as a result, the fluorescence of the PIM-1 complex is quenched. This is, however, not observed in the case of the BN analyte molecule or the acetonitrile control. The LUMO orbital energy of BN is −0.10 eV, which is higher than the LUMO of PIM-1 (−2.20 eV). This prohibits the formation of a charge-transfer state between PIM-1 and BN. Furthermore, calculations of the HOMO and LUMO orbitals of the complexes 1U-PIM-1 + DNT and 1U-PIM-1 + TNT show that the LUMO is located on the DNT and TNT, respectively, while the HOMO is on 1U-PIM-1. In contrast, the LUMO orbital of the complex 1U-PIM-1 + BN remains unchanged compared with the LUMO of an isolated 1U-PIM-1 (Figure 3b and Section SI 6).

**Figure 3:**
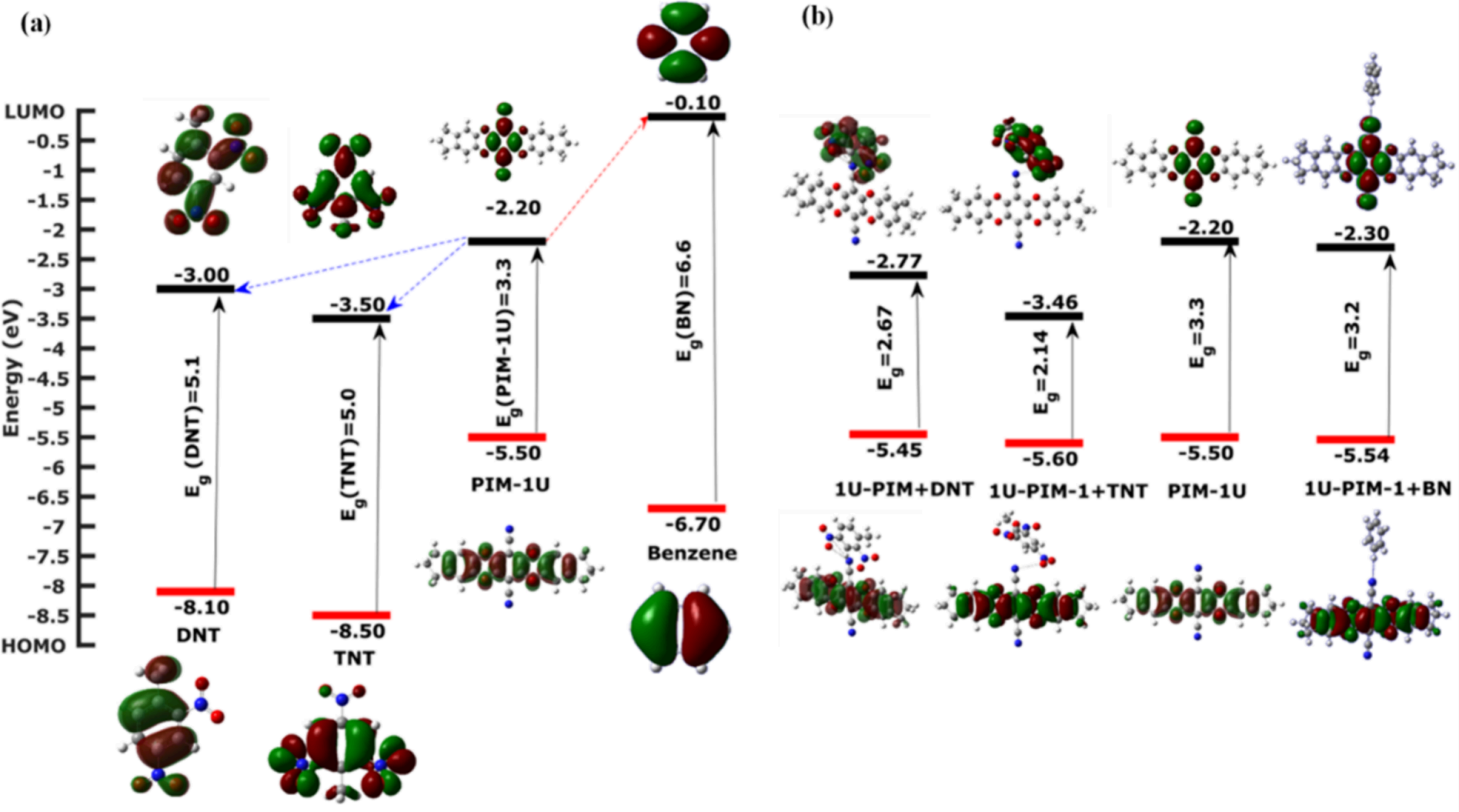
(a) Frontier molecular orbitals of HOMO and LUMO of DNT, TNT, 1U-PIM-1, and BN. (b) Frontier molecular orbitals of HOMO and LUMO of 1U-PIM-1 + DNT, 1U-PIM-1 + TNT, and 1U-PIM-1 + BN.

We next consider the changes relating to a strong noncovalent binding interaction between PIM-1 and, e.g., DNT.

Figure 4a shows the change in the absorbance of the film due to 16.39 μg of DNT dispersed in a PIM-1 film of thickness 180 nm (obtained after subtracting the absorbance due to PIM-1) and the subsequent thermal desorption of the DNT following the heating procedure described in the Methods section. By measuring the change in absorbance at 241 nm during thermal desorption of DNT, we can estimate the desorption energy *E*_d_ by applying the modified Arrhenius equation (eq 10) to the experimental data in Figure 4a. The observed reduction in the absorbance peaks at 241 nm can be attributed to DNT molecules being released from the film after each heating step. The DNT absorbance remains approximately constant until the film temperature is increased to 70 °C. The drop in absorbance is initially relatively small, even when heated at 100 °C, indicating the strong binding interaction between DNT and PIM-1, which can only be overcome at higher temperatures.

**Figure 4:**
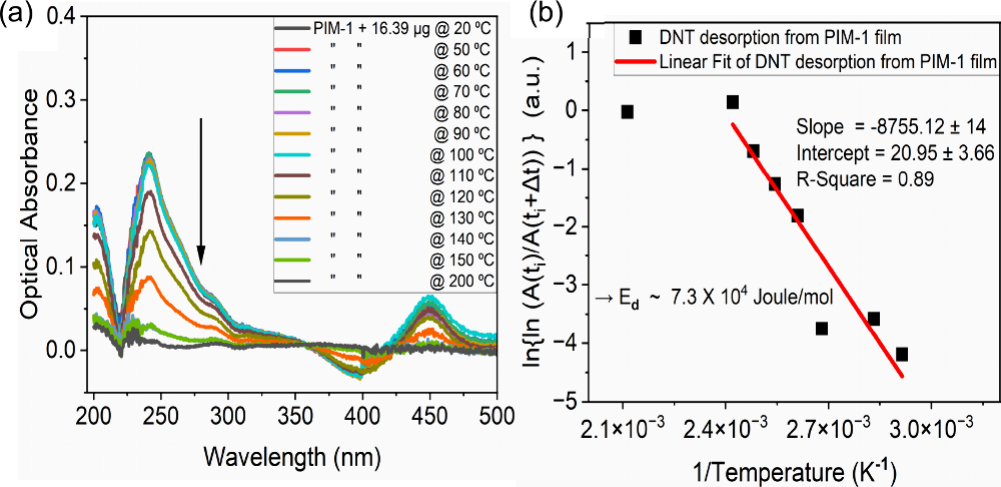
Thermal desorption of DNT from the PIM-1 film: (a) Optical absorbance of 16.39 μg of DNT in the PIM-1 matrix, obtained after subtracting the absorbance due to pristine PIM-1, and subsequent drop in the absorbance with an increase in temperature from 50 to 200 °C. (b) Natural log of change in absorbance against the inverse of temperature for DNT in the PIM-1 film.

The negative and positive peaks at 400 and 450 nm correspond to the spectral shift of this PIM-1 absorption band in the presence of DNT. We note that these peaks, and hence the population of bound charge-transfer states, reduce simultaneously with the reduction in the DNT absorption band (240–300 nm), confirming that the desorbed DNT molecules are released from binding sites within the PIM-1 matrix. These peaks disappear when the film is heated to 200 °C, indicating that the PIM-1 film returns to its pristine state following desorption. Figure 4b shows a graph of the modified Arrhenius plot (eq 10), used to estimate the *E*_d_ of the DNT thermal desorption data of Figure 4a. A linear fit to the absorbance data obtained from 70 to 140 °C (corresponding to 2.9 × 10^–3^ to 2.4 × 10^–3^ K^–1^ on the 1/*T*-axis, respectively) gives a desorption energy of 73 kJ/mol.

We also calculated the binding energy (Δ*E*), applying the B3LYP-gCP-D3/6-31G­(d) functional, for PIM-1 in interaction with DNT, using four different configurations of the molecular model (1U-PIM-1 + DNT, 2U-PIM-1 + DNT, 3U-PIM-1 + DNT, and 10U-PIM-1 + DNT), as shown in Section SI 7.

The representations of calculated Δ*E* and the corresponding measured values of desorption energy are shown in Table 1 and Figure 5. The calculated values of Δ*E* for 1U-PIM-1 + DNT, 2U-PIM + DNT, 3U-PIM-1 + DNT, and 10U-PIM-1 + DNT are 30.9, 42.6, 68.2, and 75.8 kJ/mol, respectively. The reference point (Δ*E* = 0) represents a noninteracting case. For comparison, we calculated also Δ*E* for three configurations of PIM-1 (1U-PIM-1, 2U-PIM-1, and 3U-PIM-1) attached to TNT and BN. The data in Table 1 shows that the Δ*E* results for TNT (31.5, 41.7, and 67.9 kJ/mol) are similar to the corresponding DNT results. In contrast, however, the calculated values of Δ*E* for BN are much smaller (7.9, 20.9, and 32.7 kJ/mol) than those of DNT and TNT. The stabilized configuration of the complex indicates that the DNT molecule causes a bending of the rigid planar PIM-1 chain (3U-PIM-1), arising from the Coulomb interaction between the polymer and DNT (see Section SI 8). We note that the Δ*E* value may vary with the relative orientation of 1U-PIM-1 and the interacting molecules. Therefore, we also considered a second possible configuration of 1U-PIM-1 + DNT. The calculated Δ*E* for this configuration is 15.9 kJ/mol, compared to 30.9 kJ/mol for the head-to-head configuration. These results indicate that the head-to-head interaction between DNT and 1U-PIM-1 is more stable than the coplanar configuration (Section SI 9).

**Table 1:** Calculated Binding Energies (Δ*E*) for Various Configurations of XU-PIM-1 Interacting with DNT, TNT, and BN^a^

	Δ*E* (kJ/mol), **B3-LYP-gCP-D3(0)/6-31G(d)**		
configurations	**DNT**	**TNT**	**BN**
cal. (1U-PIM-1)	30.9	31.5	7.9
cal. (2U-PIM-1)	42.6	41.7	20.9
cal. (3U-PIM-1)	68.2	67.9	32.7
cal. (10U-PIM-1)	75.8	–	32.8
exp. (PIM-1 + DNT)	73.0	–	–

a The Δ*E* values were computed by using B3LYP-gCP-D3(0)/6-31G­(d) methods. For comparison, the experimentally determined binding energy of the PIM-1 + DNT complex is also included.

**Figure 5:**
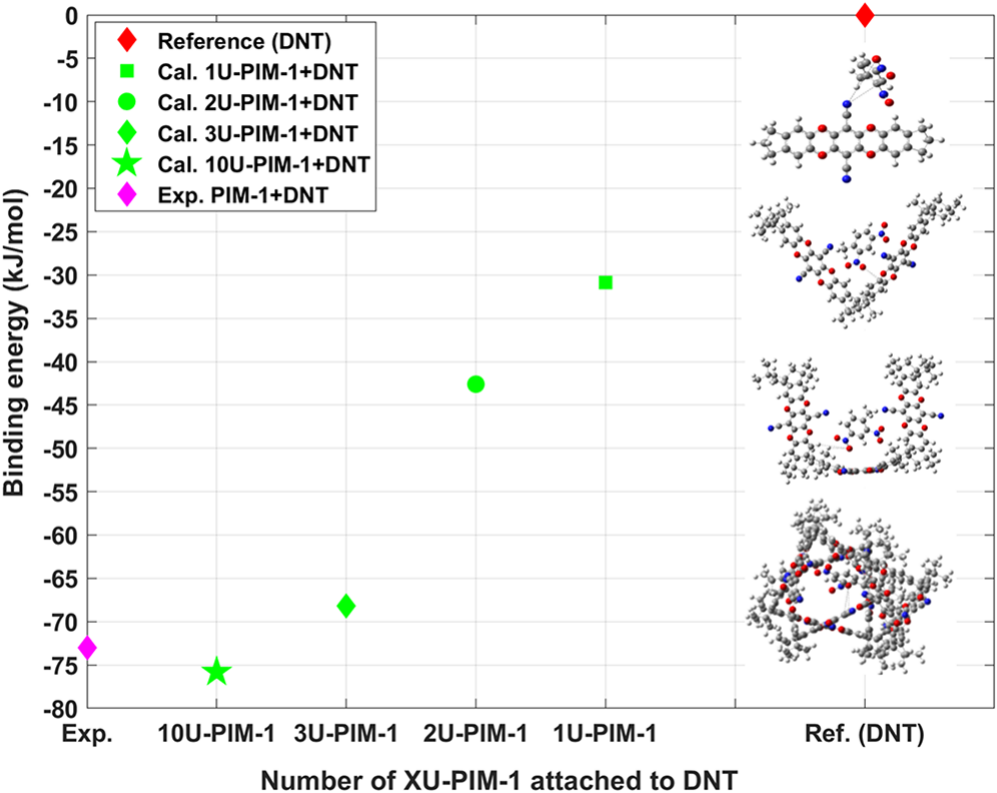
Calculated Δ*E* values for the interaction between DNT and four different configurations of PIM-1: 1U-PIM-1 + DNT, 2U-PIM-1 + DNT, 3U-PIM-1 + DNT, and 10U-PIM-1 + DNT. The calculation is based on the B3LYP-gCP-D3/6-31G­(d) functional. Lower Δ*E* values indicate weaker binding interactions. Among the configurations, 10U-PIM-1 + DNT exhibits the strongest interaction with a Δ*E* of 75.8 kJ/mol. Experimental binding energy, estimated from absorbance changes at 241 nm during thermal desorption of DNT, is 73 kJ/mol (indicated in pink), showing an excellent agreement with the predicted value.

In accordance with the discussion above, it is clear (see Table 1 and Figure 5) that the dominant values of Δ*E* are obtained from the configuration of the 10U-PIM-1 + DNT complex (75.8 kJ/mol). This value is more than twice that of the BN complex (32.8 kJ/mol). A detailed analysis of the Δ*E* for the 3U-PIM-1 + DNT complex reveals that the energy difference between the 2U-PIM-1 + DNT and 3U-PIM-1 + DNT complexes is 25.6 kJ/mol, whereas the difference between the 3U-PIM-1 + DNT and 10U-PIM-1 + DNT complexes is 7.6 kJ/mol. These findings indicate that the Δ*E* value for the 3U-PIM-1 + DNT complex converges more rapidly toward that of the 10U-PIM-1 + DNT complex than it does from 2U-PIM-1 + DNT to 3U-PIM-1 + DNT. The result shows also that the 3U-PIM-1 + DNT already brings us close to the experimental value for the binding energy, and extending to 10U-PIM-1 + DNT makes a further small correction that is much less than the change from 2U-PIM-1 + DNT to 3U-PIM-1 + DNT. A key finding of this work is that while accurate modeling of binding sites and interaction energies ideally requires a representation that captures the polymeric nature of PIM-1, relatively small molecular fragments can still reproduce the experimentally observed trends in electronic properties with high fidelity. This case study highlights the utility of molecular modeling as a practical tool to strike a balance between computational cost and predictive power, offering valuable guidance for interpreting and designing experiments.

Our results indicate that the nitroaromatic molecules are electrostatically bound in pockets formed by three repeat units of the polymer. These pockets in PIM-1 arise due to the twisted conformation of the spiro linkage between adjacent repeat units of the polymer chain. These microscopic structures are subject to thermal motions within and between the polymer chains and so are difficult to control; however, the binding of the nitroaromatic molecule apparently modifies the local conformation by bending the planar sections of the pocket.

Absorption of solvent/analyte molecules can also change the packing of adjacent chains by swelling the polymer matrix. This additional free volume is reversible, and on removal of the solvent, the PIM film returns to its original denser state. In our experiments, we observe the acetonitrile solvent to be desorbed at room temperature from these 180 nm thick films on a 5 min time scale, returning the film to its original state. Absorbed DNT molecules are more strongly bound in the trimer pockets in the film, but we find that heating to remove DNT molecules causes the shifted optical absorption band around 420 nm to return to that of the original pristine polymer, indicating that the polymer reverts to its original configuration.

## Conclusions

A motivation for studying explosive analytes at the molecular level is that it renders the possibility to identify the quenching process for detection of explosive substances at ultralow concentrations. In such applications, changes in the intensity of emission can be used for real-time or off-line sensing. To summarize the finding of the present study, we find that the addition of the nitroaromatic analyte leads to a red shift of the PIM-1 absorption band around 420 nm and that addition of DNT and TNT causes a greater shift than that observed for PIM-1 + BN. The calculated spectra and changes in transition oscillator strength are in good agreement with the corresponding experimental results. The quenching of the emission can be explained by an excited-state electron transfer from PIM-1 to DNT. The calculated frontier molecular orbitals in the composite 1U-PIM + DNT or 1U-PIM + TNT structures show the highest occupied molecular orbital to be located on a planar section of the PIM-1 backbone in each case, while the lowest unoccupied molecular orbital lies on the nitroaromatic analyte. Exposure to DNT or TNT also leads to a strong quenching of the fluorescence but without a significant change in the spectrum. These changes relate to a strong noncovalent binding interaction between PIM-1 and DNT or TNT; we calculate the binding energy, using the B3LYP-gCP-D3/6-31G­(d) functional, to be 75.8 kJ/mol for the interaction of DNT with a 10-unit oligomer of PIM-1. By measuring the changes in absorbance at 230 nm during thermal desorption of DNT, we estimate a binding energy of 73 kJ/mol, in excellent agreement with the calculation.

Molecular interactions typically require a quantum-based approach, which places high demands on both the scalability and the accuracy of the applied methodology. In this context, a key finding of this work is that while accurate calculations of molecular binding sites (and their corresponding strengths) generally require models that capture the full polymeric nature of PIM-1, we demonstrate that a smaller molecular model, comprising just a single repeat unit fragment of PIM-1, can effectively replicate the experimentally observed changes in electronic properties. This case study highlights the potential of molecular modeling to predict experimental outcomes with both flexibility and precision. The study could be extended in the future also to quantify the energetics of electron transfer by expanding the simulation approach to include the hybrid quantum mechanics/molecular mechanics (QM/MM) level of theory.

These findings indicate also that PIM-1 holds significant promise as a sensor material for explosives. The observed molecular interactions indicate that analyte molecules can dock in a trimer pocket of the highly contorted backbone of the polymer. This mechanistic insight into the binding interaction of the trimer of PIM-1 with DNT and TNT could be used to help guide the molecular design of other microporous polymers for fluorescence-based sensing. Continued investigation into these interactions may ultimately enable the design of more selective sensors capable of distinguishing among various explosive compounds, thereby enhancing detection accuracy.

## Supplementary Material



## Data Availability

The research data supporting this publication^37^ can be accessed at https://10.17630/98a379d9-93a6-494a-9b08-d9d2ec4caf1e.

## Notes

Synthesis procedure for PIM-1; optimized molecular structure for interactions between PIM-1 and analytes; optical absorption of 2,4-DNT and 2,4,6-TNT in acetonitrile solution; photoluminescence of PIM-1 and PL quenching due to analytes; molecular electrostatic potentials of 1U-PIM-1 and interactions with the analytes; frontier molecular orbitals in the composite 1U-PIM-1 and each analyte; optimized molecular configurations used for calculations of the binding energy; molecular electrostatic potentials showing how the rigid planar PIM-1 unit bends due to DNT binding; calculated binding energy for head-to-head and coplanar binding of 1U-PIM-1 + DNT; calculated binding energy for 3U-PIM-1 + DNT; calculated binding energy for 3U-PIM-1 + TNT; calculated binding energy for 3U-PIM-1 + BN; calculated binding energy for 10U-PIM-1 + DNT; calculated binding energy for 10U-PIM-1 + BN; and the summary of calculated binding energies for the interaction between DNT and four different configurations of PIM-1: 1U-PIM-1 + DNT, 2U-PIM-1 + DNT, 3U-PIM-1 + DNT, and 10U-PIM-1 + DNT (PDF)
