# Supplementary material for: Interaction of Polymer of Intrinsic Microporosity PIM‑1 with Explosive Analytes at the Molecular Level: Combined Experiment and Computational Modeling
Source: J Phys Chem C Nanomater Interfaces. 2026 Apr 18;130(22):7664–71. doi: 10.1021/acs.jpcc.5c08293 (PMC13244556; doi:10.1021/acs.jpcc.5c08293)
Supplement: Supplementary file 1 [file jp5c08293_si_001.pdf]

Supporting Information

Interaction of Polymer of Intrinsic Microporosity PIM-1 with Explosive Analytes at the Molecular Level: Combined Experiment and Computational Modelling

Salam Mohammed[1,2], Edward B. Ogugu[2], Ramakant Sharma[2], Dominic Taylor[3], Graeme Cooke[4], Neil B. McKeown[3], Glib Baryshnikov[5], Hans Ågren[6,7], Ifor D.W. Samuel[2], Graham A. Turnbull[2]

1. Swedish EOD and Demining Centre-SWEDEC, Swedish Armed Forces, SE-575 28 Eksjö, Sweden.
2. Organic Semiconductor Centre, School of Physics and Astronomy, University of St Andrews, St Andrews, KY16 9SS, UK.
3. School of Chemistry, University of Edinburgh, Edinburgh EH9 3FJ, UK.
4. School of Chemistry, University of Glasgow, Glasgow G12 8QQ, UK.
5. Department of Science and Technology (ITN), Linköping University, SE-581 8 Linköping, Sweden.
6. Department of Physics and Astronomy, X-ray Photon Science, Uppsala University Box 516, 751 20 Uppsala, Sweden.
7. *Faculty of Chemistry, Wroclaw University of Science and Technology, Wyspianskiego 27, PL-50370 Wroclaw, Poland*

Author e-mail address: gat@st-andrews.ac.uk and salam.mohammed@mil.se

SI 1. Synthesis procedure for PIM-1

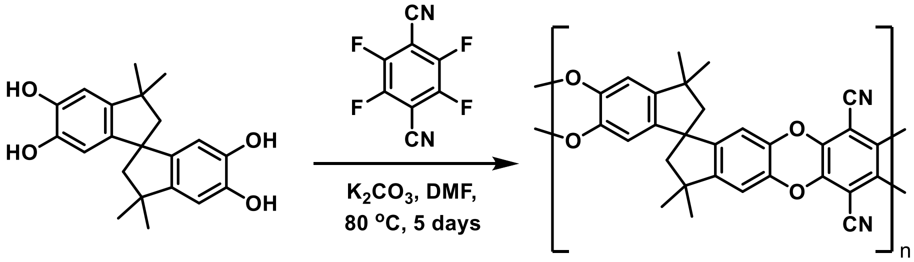

Figure S1. 5,5',6,6'-Tetrahydroxy-3,3,3',3'-tetramethyl-1,1'-spirobisindane (13.617 g, 40 mmol), tetrafluoroterephthalonitrile (8.004 g, 40 mmol) and potassium carbonate (16.59 g, 120 mmol) were suspended in DMF (150 mL) and heated to 80 °C for 5 days under a nitrogen atmosphere. After cooling, the reaction mixture was poured onto methanol (300 mL) to yield a yellow precipitate that was filtered, washed with water (1 L) and methanol (200 mL). The crude polymer was purified by two reprecipitations from chloroform (300 mL) solution into methanol (1 L). The crude product was then dried in a conventional oven for 3 days at 100 °C then a vacuum oven at 100 °C for 6 hours to yield a bright yellow powder (17.02 g, 92%). ^{1}H NMR (500 MHz, $CDCl_3$, 25.0 °C) δ_H 6.81 (bs, 2 *H*), 6.41 (bs, 2 *H*), 2.34 (bs, 2 *H*), 2.16 (bs, 2 *H*), 1.56 (s, 6 *H*), 1.37 (s, 6 *H*), 1.31 (s, 6 *H*). Data in agreement with reported spectra. Gel permeation chromatography, eluent = chloroform, calibrated against polystyrene standards: M_W = 104 870 g mol^{-1}, M_n = 17 711 g mol^{-1}, PDI = 5.92

SI 2. Optimized molecular structure for interactions between PIM-1 and analytes

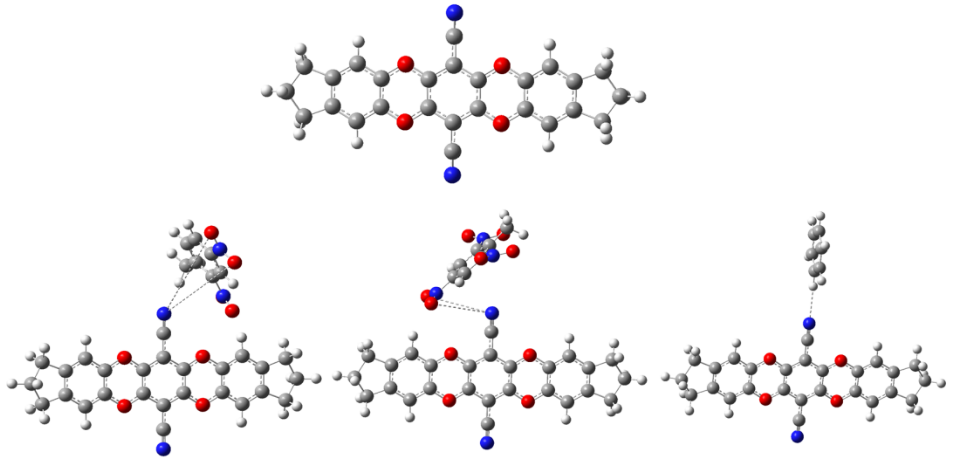

Figure S2. Optimized molecular structure (head-to-head) interaction between the CN group of 1U-1U-PIM-1 and analytes of DNT, TNT and BN, respectively.

SI 3 Optical absorption of 2,4-DNT and 2,4,6-TNT in acetonitrile solution

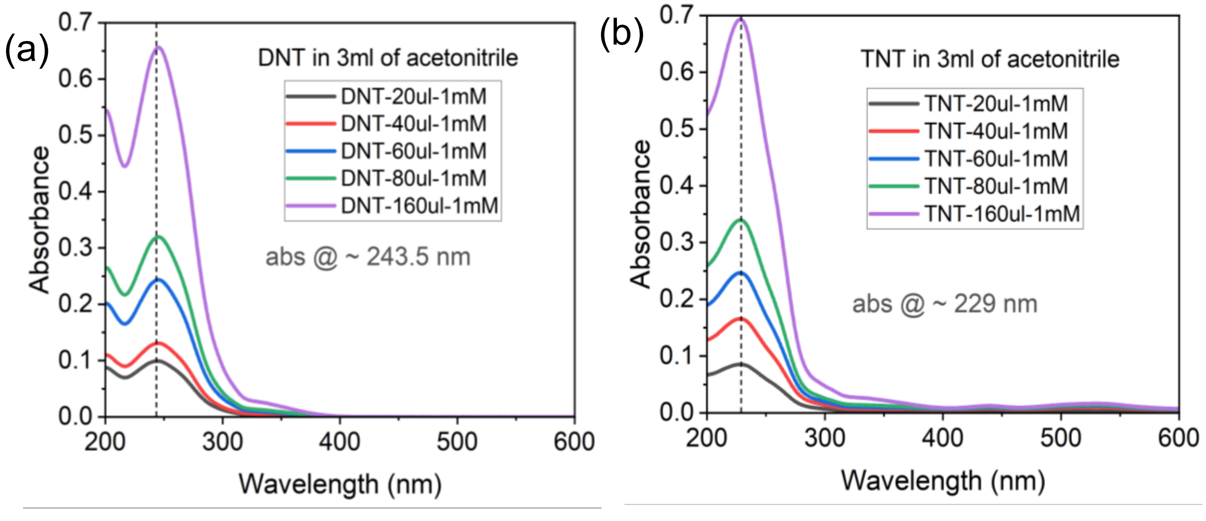

Figure S3. Optical absorption of 2,4-DNT and 2,4,6-TNT in acetonitrile solutions: (a) absorbance increase with increase in DNT concentration. (b) increase in absorbance with increasing TNT concentration.

A stock solution of DNT was prepared at a concentration of 1 mM in acetonitrile. Then, 20 µL of the 1 mM DNT solution was added to 3 mL of acetonitrile in a cuvette before absorption measurement. The procedure of adding DNT solution was repeated up to 5 aliquots, as shown in SI 3(a), yielding DNT concentrations of 3.64 µg, 7.28 µg, 10.92 µg, 14.56 µg, and 29.12 µg, respectively. A similar experiment was conducted for the TNT solution, as shown in SI 3(b), yielding concentrations of 4.54 µg, 9.09 µg, 13.63 µg, 18.17 µg, and 36.34 µg, respectively.

SI 4. Photoluminescence of PIM-1 and PL quenching due to analytes

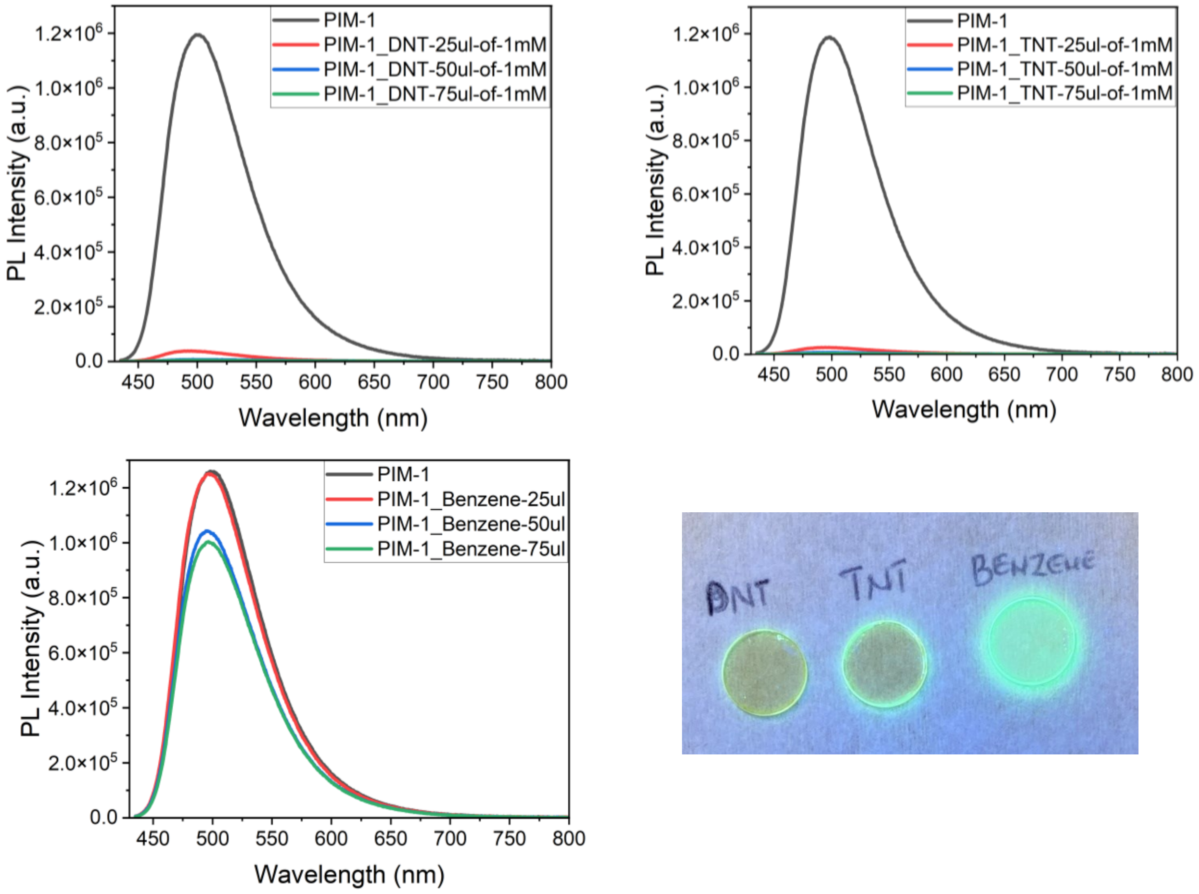

Figure S4. Photoluminescence (PL) of PIM-1 and subsequent PL quenching due analytes: (a) PL quenching of PIM due to DNT at various concentration, (b) PL quenching with increasing concentration of TNT, (c) Slight drop in the PL of PIM-1 due to benzene solvent, and (d) a photo showing PIM-1 films doped with 13.66 µg of DNT, 17.03 µg of TNT, and 75 µL of benzene.

The PIM-1 films were doped with 2,4,6-trinitrotoluene (TNT) and 2,4-dinitrotoluene (DNT) by drop-casting 25 µL of the respective solutions of 1 mM in acetonitrile onto the PIM-1 films, and

solutions were allowed to evaporate, leaving behind molecules of analytes adsorbed in the films before PL measurements. The response of PIM-1 to non-explosive molecules was also explored by drop casting 25 µL of benzene. The procedure was repeated up to three aliquots to examine the concentration of analytes, which would result in complete PL quenching of the PIM-1. The three aliquots employed resulted in concentrations of 4.55 µg, 9.11 µg, and 13.66 µg, respectively, of DNT in PIM-1, and 5.68 µg, 11.36 µg, and 17.03 µg, respectively, of TNT in PIM-1.

SI 5. Molecular electrostatic potential of 1U-PIM-1 and interactions with the analytes

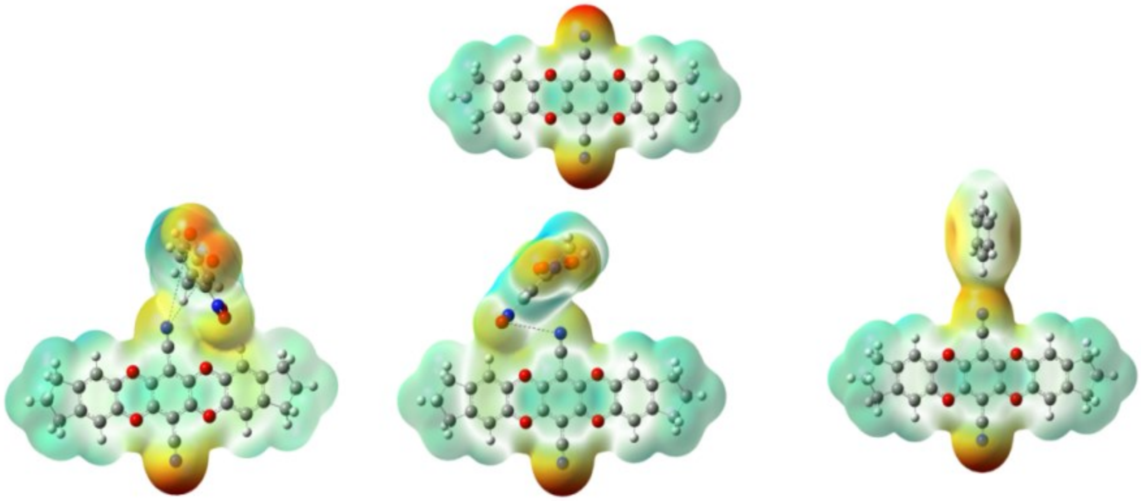

Figure S5. Molecular electrostatic potentials (MEP) maps of 1U-PIM-1 (upper) and Interactions between the CN group of 1U-PIM-1 with the analyte of DNT, TNT and BN, respectively (lower). Electron rich (negative charge) and the electron deficient (positive charge) is represented by red and blue color, respectively.

SI 6. Frontier molecular orbitals in the composite 1U-PIM-1 and each analyte

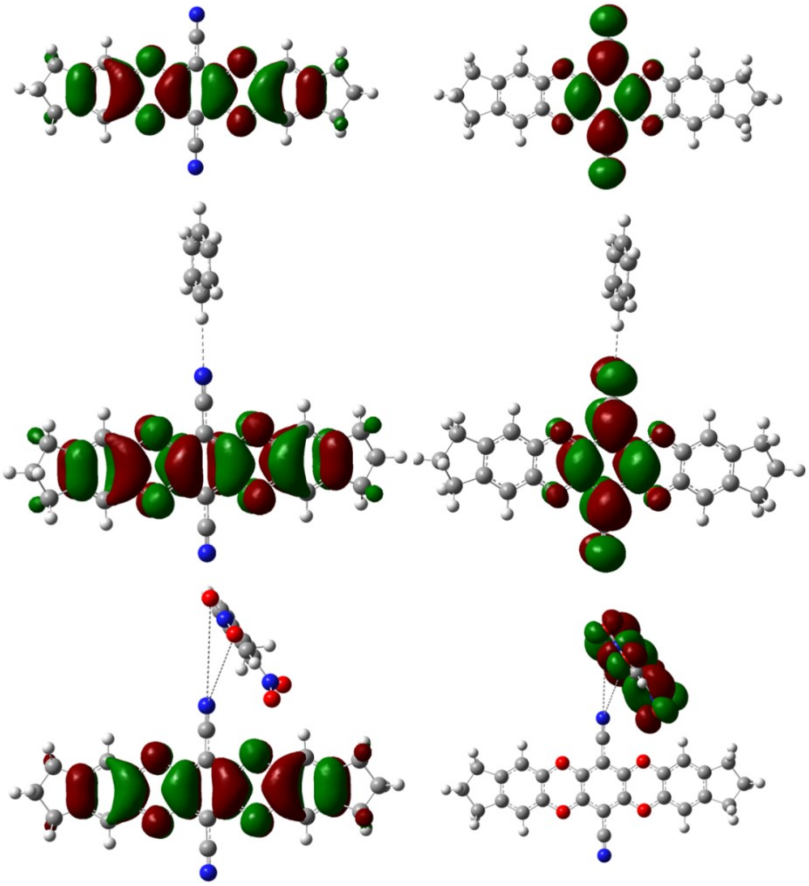

Figure S6. Calculated frontier molecular orbitals (HOMO and LUMO) in the composite 1U-PIM-1 (Upper), 1U-PIM+BN (Meddle) and1U-PIM+DNT (lower). The structures show the highest occupied molecular orbital HOMO to be located on a planar section of the 1U-PIM-1 backbone in each case, while the lowest unoccupied molecular orbital LUMO lies on the nitroaromatic analyte of DNT as the only case.

SI 7. Optimized molecular configurations used for calculations of the binding energy

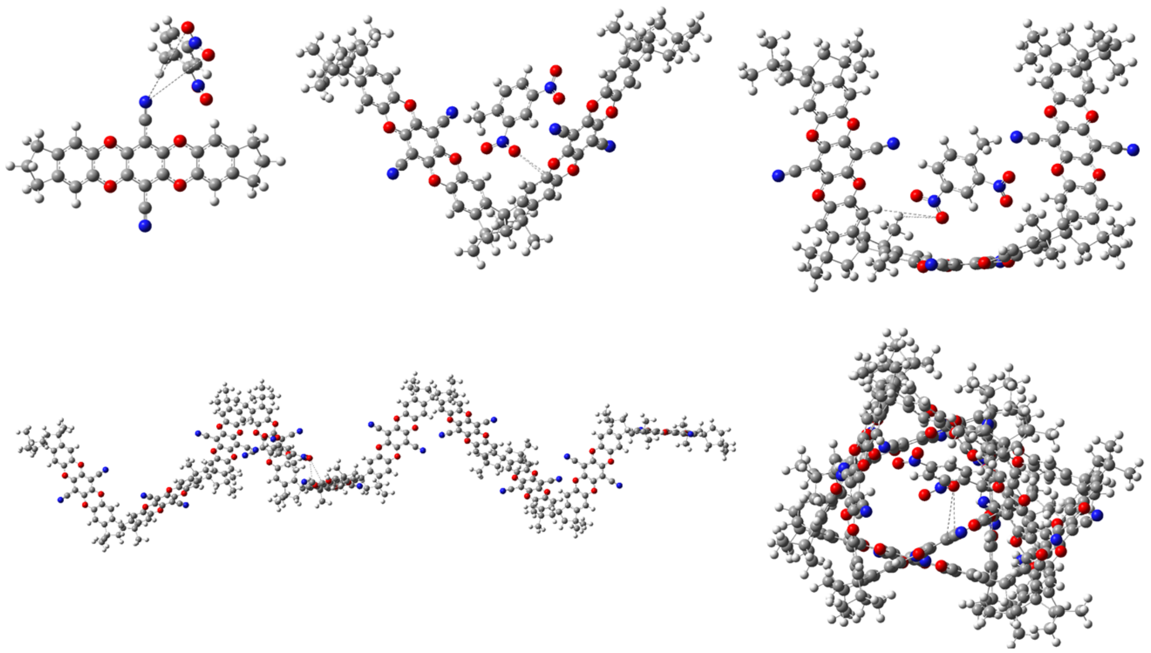

Figure S7. Four different optimized configurations were used for the calculations of the binding energy *ΔE* between PIM-1 and DNT. 1U-PIM-1+DNT (upper-left), 2U-PIM-1+DNT (upper-middle), 3U-PIM-1+DNT (upper-right) and 10U-PIM-1+DNT (lower-left: front view and lower right: the side view).

SI 8. Molecular electrostatic potential showing how the rigid planar PIM-1 unit bends due to DNT binding

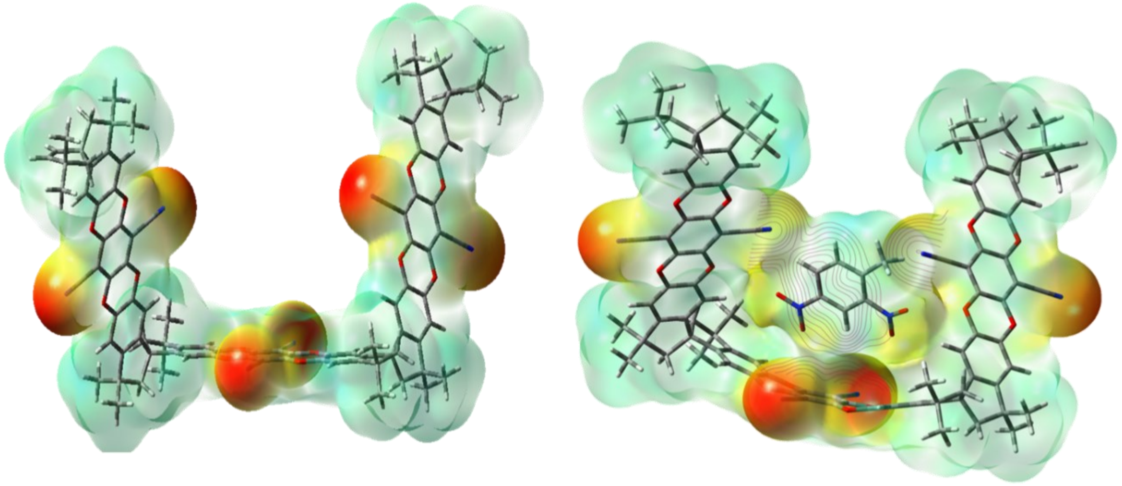

Figure S8. Molecular electrostatic potentials (MEP) maps show the DNT molecule causes a bending of the rigid planar PIM-1 chain 3U-PIM-1, arising from the coulomb interaction between the polymer and DNT (compare left: 3UPIM-1 to right:3U-PIM+DNT). Electron rich (negative charge) and the electron deficient (positive charge) is represented by red and blue color, respectively.

SI 9. Calculated binding energy for head-to-head and coplanar binding of 1U-PIM-1+DNT

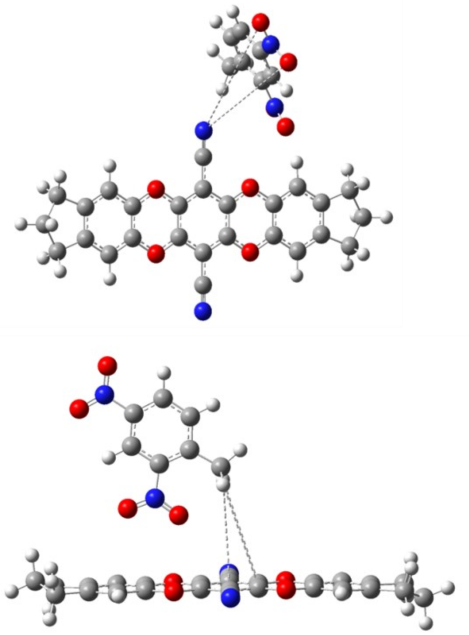

Figure S9. Calculated *ΔE* for head-to-head (upper) and coplanar (lower) of 1U-PIM-1+DNT is 30.9 and 15.9 kJ/mol, respectively.

SI 10. Calculated binding energy for 3U-PIM-1+DNT

Calculated the ΔE (kJ/mol) for 3U-PIM+DNT using B3LYP-D3-gCP/6-31G(d)/ 6-31g(d)

<u>DNT: (a.u.)</u>

SCF energy -680.561825657

gCP correction 0.0456961305

D3 correction -0.01627335

gCP-D3 correction 0.0294227805

SCF-gCP-D3 energy -680.5324028765

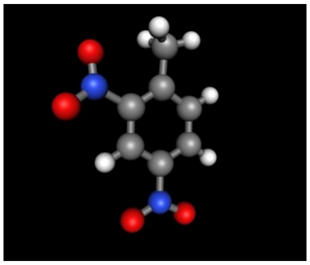

<u>3U-PIM (a.u.)</u>

SCF energy -5089.88617953

gCP correction 0.4602602174

D3 correction -0.25417230

gCP-D3 correction 0.2060879174

SCF-gCP-D3 energy -5089.6800916126

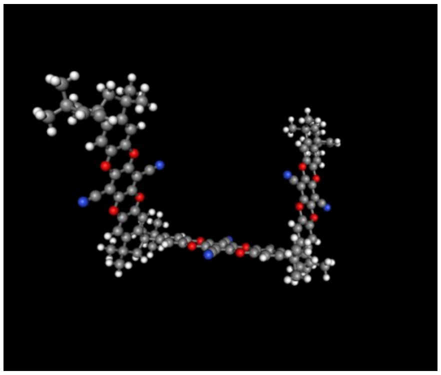

<u>3U-PIM+DNT (a.u)</u>

SCF energy -5770.46445349

gCP correction 0.5169216454

D3 correction -0.29092635

gCP-D3 correction 0.2259952954

SCF-gCP-D3 energy -5770.2384581946

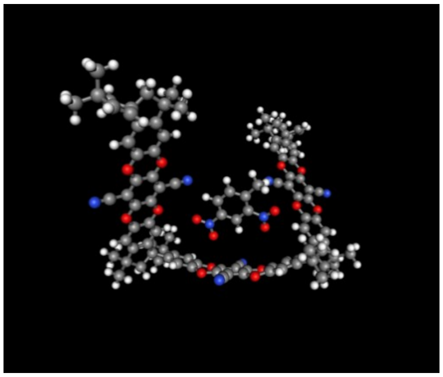

Cal. $(\Delta E)_{3U\text{-}PIM+DNT}$ = - 68.2 kJ/mol,

Exp. $(\Delta E)_{PIM\text{-}1+DNT}$ = 73 kJ/mol

SI 11. Calculated binding energy for 3U-PIM-1+TNT

Calculated the ΔE (kJ/mol) for 3U-PIM+TNT using B3LYP-D3-gCP/6-31G(d)/ 6-31g(d)

<u>**TNT (a.u.):**</u>

SCF energy -885.045483764

gCP correction 0.0545468025

D3 correction -0.02132730

gCP-D3 correction 0.0332195025

SCF-gCP-D3 energy -885.0122642615

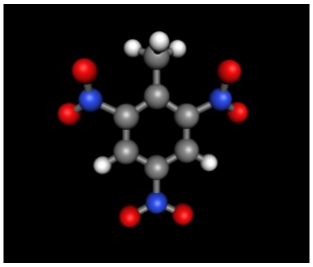

<u>**3U-PIM (a.u.)**</u>

SCF energy -5089.88617953

gCP correction 0.4602602174

D3 correction -0.25417230

gCP-D3 correction 0.2060879174

SCF-gCP-D3 energy -5089.6800916126

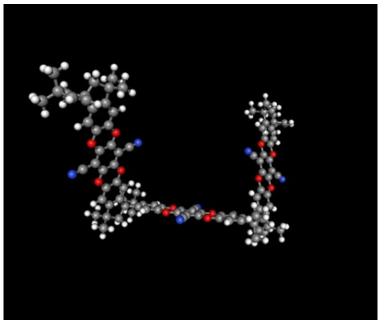

<u>**3U-PIM-1+TNT(a.u.)**</u>

SCF energy -5974.94746478

gCP correction 0.5261230893

D3 correction -0.29686098

gCP-D3 correction 0.2292621093

SCF-gCP-D3 energy -5974.7182026707

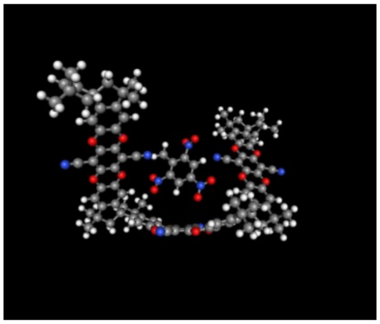

Cal. (ΔE) $_{\text{3U-PIM+TNT}}$ = - 67.86 kJ/mol

Cal. (ΔE) $_{\text{3U-PIM+DNT}}$ = - 68.2 kJ/mol,

Exp. (ΔE) $_{\text{PIM-1+DNT}}$ = 73 kJ/mol

SI 12. Calculated binding energy for 3U-PIM-1+BN

Calculated the *ΔE* (kJ/mol) for 3U-PIM+BN using B3LYP-D3-gCP/6-31G(d)/ 6-31g(d)

<u>**BN:**</u>

SCF energy -232.248649431

gCP correction 0.0220835213

D3 correction -0.00509906

gCP-D3 correction 0.0169844613

SCF-gCP-D3 energy -232.2316649697

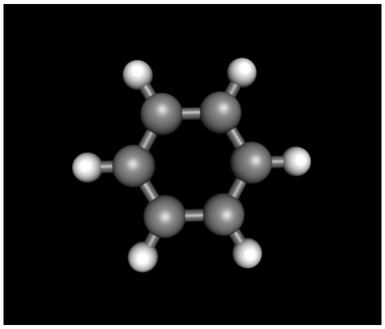

<u>**3U-PIM**</u>

SCF energy -5089.88617953

gCP correction 0.4602602174

D3 correction -0.25417230

gCP-D3 correction 0.2060879174

SCF-gCP-D3 energy -5089.6800916126

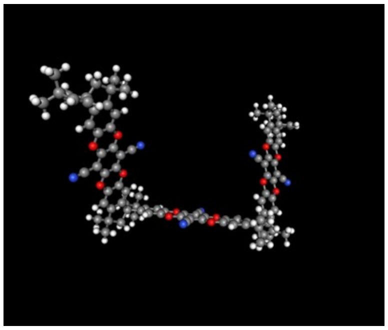

<u>**3U-PIM+BN**</u>

SCF energy -5322.13887480

gCP correction 0.4848322965

D3 correction -0.27018913

gCP-D3 correction 0.2146431665

SCF-gCP-D3 energy -5321.9242316335

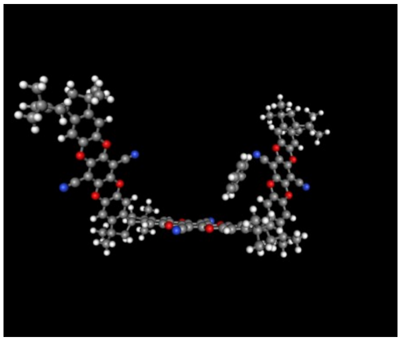

Cal. (ΔE) $_{3U\text{-}PIM+BN}$ = -32.75 kJ/mol

Cal. (ΔE) $_{3U\text{-}PIM+TNT}$ = - 67.86 kJ/mol

Cal. (ΔE) $_{3U\text{-}PIM+DNT}$ = - 68.2 kJ/mol,

Exp. (ΔE) $_{PIM\text{-}1+DNT}$ = 73 kJ/mol

SI 13. Calculated binding energy for 10U-PIM-1+DNT

Calculated the BE of ΔE (kJ/mol) for 10U-PIM+DNT using B3LYP-D3-gCP/6-31G(d)/ 6-31g(d)

<u>**DNT (a.u.):**</u>

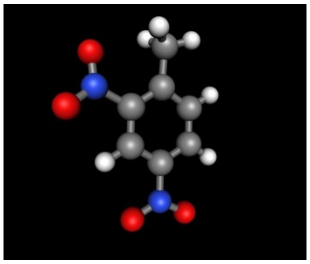

SCF energy -680.561825657

gCP correction 0.0456961305

D3 correction -0.01627335

gCP-D3 correction 0.0294227805

SCF-gCP-D3 energy -680.5324028765

<u>**10U-PIM(a.u.)**</u>

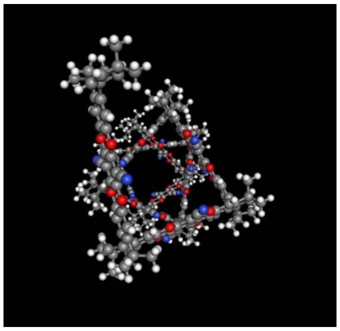

SCF energy -15782.347172
gCP correction1.3589258086
D3 correction -0.7773596
gCP-D3 correction 0.5815662086
SCF-gCP-D3 energy -15781.7656057914

<u>**10U-PIM+DNT(a.u.)**</u>

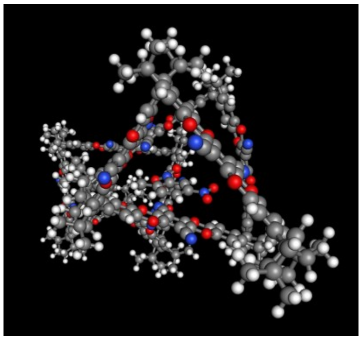

SCF energy -16462.922157983
gCP correction 1.4193129541
D3 correction - 0.8240356170
gCP-D3 correction 0.5952773371
SCF-gCP-D3 energy -16462.3268806459

Cal. (ΔE) $_{\text{10U-PIM-1+DNT}}$ = - 75.8 kJ/mol,

Exp. (ΔE) $_{\text{PIM-1+DNT}}$ = 73 kJ/mol

SI 14. Calculated binding energy for 10U-PIM-1+BN

Calculated the BE of *ΔE* (kJ/mol) for 10U-PIM+BN using B3LYP-D3-gCP/6-31G(d)/ 6-31g(d)

<u>**BN:**</u>

SCF energy -232.248649431

gCP correction 0.0220835213

D3 correction -0.00509906

gCP-D3 correction 0.0169844613

SCF-gCP-D3 energy -232.2316649697

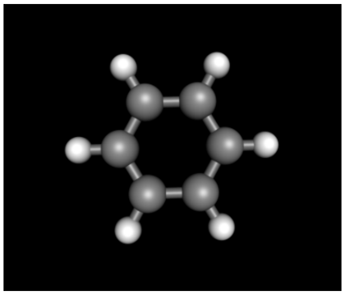

<u>**10U-PIM(a.u.)**</u>

SCF energy -15782.347172
gCP correction 1.3589258086
D3 correction -0.7773596
gCP-D3 correction 0.5815662086
SCF-gCP-D3 energy -15781.7656057914

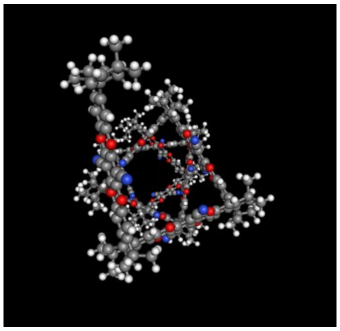

<u>**10U-PIM+BN (a.u.)**</u>

SCF energy -16015.3910506 -16,014.6048629003

gCP correction 1.3812964564

D3 correction -0.7861876997

gCP-D3 correction 1.3812964564

SCF-gCP-D3 energy -16014.0097541436

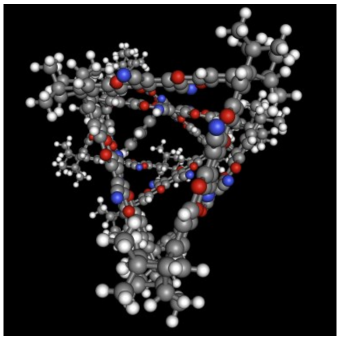

Cal. (ΔE) $_{\text{10U-PIM-1+BN}}$ =-32.8 kJ/mol

Cal. (ΔE) $_{\text{10U-PIM-1+DNT}}$ = - 75.8 kJ/mol,

Exp. (ΔE) $_{\text{PIM-1+DNT}}$ = 73 kJ/mol

SI 15. Summary of calculated binding energies for the interaction between DNT and four different configurations of PIM-1:

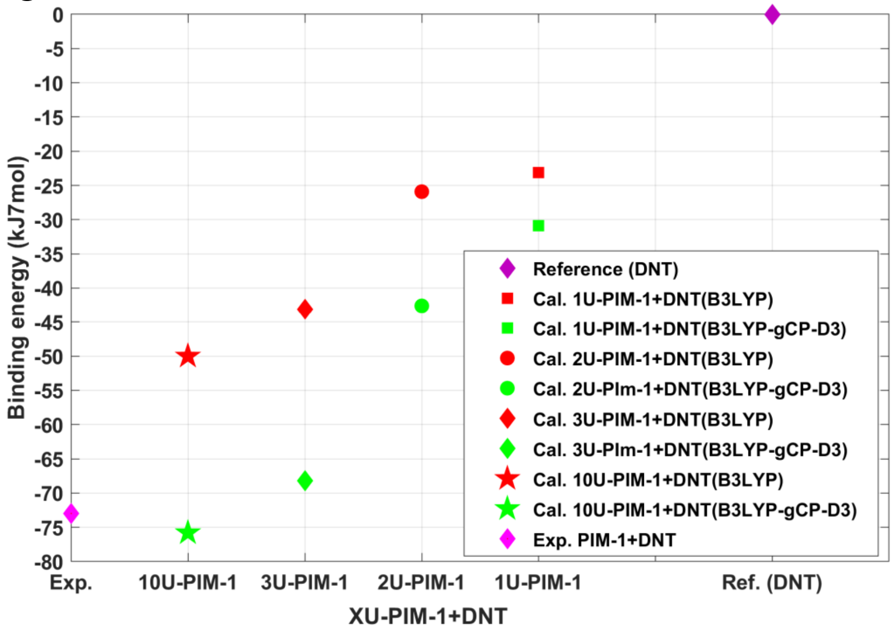

Figure S15. Calculated *ΔE* values for the interaction between DNT and four different configurations of PIM-1: 1U-PIM-1+DNT, 2U-PIM-1+DNT, 3U-PIM-1+DNT, and 10U-PIM-1+DNT. Lower *ΔE* values indicate weaker binding interactions. Results from standard B3LYP calculations are shown in red, while values obtained using B3LYP with gCP-D3 corrections are shown in green. Among the configurations, 10U-PIM-1+DNT exhibits the strongest interaction, with a *ΔE* of 75.8 kJ/mol. Experimental binding energy, estimated from absorbance changes at 241 nm during thermal desorption of DNT, is 73 kJ/mol (indicated in pink), showing good agreement with the corrected theoretical value.

Table S1. Calculated binding energies (*ΔE*) for various configurations of XU-PIM-1 interacting with DNT, TNT, and BE**.** The *ΔE* values were computed using both B3LYP/6-31G(d) and B3LYP-gCP-D3/6-31G(d) methods. For comparison, the experimentally determined binding energy of the PIM-1+DNT complex is also included.

**Configurations**	**ΔE (kJ/mol) B-3LYP/B3-LYP-gCP-D3(0)/6-31g(d)**		
	**DNT**	**TNT**	**BN**
**Cal. (1U-PIM-1)**	**23.1/ 30.9**	**23.1/ 31.5**	**5.8/ 7.9**
**Cal. (2U-PIM-1)**	**25.9/ 42.6**	**25.3/ 41.7**	**6.1/ 20.9**
**Cal. (3U-PIM-1)**	**43.1/ 68.2**	**41.5/ 67.9**	**10.6/ 32.7**
**Cal. (10U-PIM-1)**	**50.0/75.8**	**45.2/**	**25.5/32.8**
**Exp. (PIM-1+DNT)**	**73.0**	**–**	**–**
